# Diagnostic performance of *Leishmania braziliensis* and *Leishmania peruviana* antigens in the immunoblot method for the detection of american tegumentary leishmaniasis

**DOI:** 10.17843/rpmesp.2024.413.13231

**Published:** 2024-08-28

**Authors:** Nyshon Rojas-Palomino, Aidé Sandoval-Juarez, Gilmer Solis-Sánchez, Gloria Minaya-Gómez

**Affiliations:** 1 National Public Health Center, National Institute of Health, Lima, Peru. National Public Health Center National Institute of Health Lima Peru; 2 National Center for Food, Nutrition and Healthy Living, National Institute of Health, Lima, Peru. National Center for Food Nutrition and Healthy Living National Institute of Health Lima Peru

**Keywords:** Leishmaniasis, Leishmania, Western Blotting, Leishmania braziliensis, Antigens, Sensitivity and Specificity

## Abstract

This study aimed to determine the performance of Leishmania braziliensis and Leishmania peruviana antigens in the detection of ATL by using serum samples obtained between 2013 - 2016. The obtained soluble and excretion/secretion antigens were transferred to membrane nitrocellulose by immunoblot assay. The evaluation was carried out against sera confirmed for ATL, at a confidence level of 95%, determining that the soluble antigen of Leishmania braziliensis had a sensitivity of 87.7%, specificity of 100% and area under the curve of 0.95; on the other hand, Leishmania peruviana showed values of 92.3%, 95.7% and 0.94, respectively. According to the results, we recommend that the reported immunogenic regions should be characterized and analyzed in order to continue with the development of recombinant and synthetic proteins, aimed at improving the efficiency of the serological diagnosis of the disease.

## INTRODUCTION

American tegumentary leishmaniasis (ATL), a disease caused by more than 20 species of *Leishmania*, groups a set of clinical manifestations ranging from single to multiple lesions, nodular, plaque-like, among others, to lesions that may involve mucous membranes. According to the World Health Organization (WHO), our continent reports more than 1 million cases, with Brazil, Colombia and Peru together accounting for 72% of all cases of ATL ^1^^,^^2^.

In Peru, ATL is caused by 8 species, being *Leishmania braziliensis* of major importance due to the damage it can inflict on the patient, from the localized and disseminated cutaneous form to the mucosal form. Other reported species are *Leishmania peruviana*, *Leishmania guyanensis*, *Leishmania panamensis*, *Leishmania shawi*, *Leishmania lainsoni*, *Leishmania naiffi* and *Leishmania amazonensis*^3^. This disease is reported in 19 regions of the country, where more than 156,000 cases have been recorded, of which approximately 10,000 (6.4%) correspond to the mucosal form ^3^^,^^4^.

This disease is mainly diagnosed by parasitological methods such as Direct Microscopic Examination (DME) with Giemsa staining and *in vitro* culture, both confirm the disease by microscopic visualization of the parasite, these methods have high specificity, but limited sensitivity mainly due to the clinical form and the time of evolution of the disease, it is not recommended in patients with chronic skin lesions or mucosal involvement, due to the low number of amastigotes in the lesion, which hinders its detection ^5^^,^^6^. Other factors are over-aggregated bacterial infection, lack of experience in obtaining the sample and recognition of the amastigote forms ^5^^,^^7^. 

Serological methods such as Indirect Immunofluorescence (IIF) and ELISA, allow diagnosis by detection of anti-Leishmania antibodies, mainly in patients with chronic skin lesions with clinical suspicion and in those with mucosal involvement ^8^. In Peru, IIF with complete parasite as antigen is widely used in diagnosis, despite the limitations associated with cross-reactivity mainly against Chagas disease due to the considerable genomic and proteomic similarity between *Leishmania* spp and *Trypanosoma cruzi*^9^^,^^10^.

Likewise, the efficiency of serological methods is linked to the nature of the antigen, structure and location of the used protein, origin of the antigen, species or stage from which it was obtained, among others; the protein miscellany resulting from the parasite’s gene expression affects the diagnostic performance of the method ^11^^,^^12^.

Therefore, studies related to the identification and evaluation of serological biomarkers are currently being developed to improve the efficiency of the method ^13^^,^^14^, reducing inter- and intra-species variability and even the immune response by the infecting species ^15^.

In this regard, this study aimed to evaluate and determine the diagnostic performance of soluble and excretion/secretion antigens of *Leishmania braziliensis* and *Leishmania peruviana* on Immunoblot against sera from confirmed patients for the detection of American tegumentary Leishmaniasis.

**KEY MESSAGES**

Motivation for the study. To contribute to the immunogenic character of soluble and excretion/secretion antigens of *Leishmania braziliensis* and *Leishmania peruviana* with the aim of identifying proteins with diagnostic potential.

Main findings. The soluble antigen of *Leishmania braziliensis* has a sensitivity in the detection of ATL of 87.7%, specificity of 100% and a false positive rate of 20% against sera from patients with Chagas disease and 8.3% with mycosis.

Implications. Immunoblot can improve the resolution capacity in the serological diagnosis of American tegumentary Leishmaniasis, particularly in patients where the length of the disease and the clinical form make difficult the diagnosis by parasitological methods.

## THE STUDY

### Study design

An observational study of diagnostic tests was conducted at the National Referral Laboratory for Metaxenics and Parasitic Zoonoses (LRN MEZOP) of the National Institute of Health between 2013 - 2016, approved by the Research and Ethics Committee of the National Institute of Health with RD N°170-2013-DG-OGITT-OPE/INS.

### Population and sample

The study population consisted of sera stored in the LRN MEZOP-INS serum collection obtained in the framework of specialized diagnostic activities of ATL between 2013-2016.

The sample size was determined using the Epidat v3.0 program considering an expected specificity of 98%, 5% precision, healthy/sick ratio of 0.25; at a confidence level of 95%.

A total of 187 serum samples were randomly selected considering the DME method as eligibility criteria for the cutaneous form and the epidemiological clinical history in addition to the IIF result for the mucosal form.

The sample consisted of 100 sera of the cutaneous form and 30 of the mucosal form, confirmed by microscopic visualization of the amastigotes forms in DME or by indirect immunofluorescence plus the epidemiological clinical history, respectively; as well as 30 sera from healthy patients without clinical suspicion of the disease from non-endemic areas; and 27 samples of other pathologies involved in the differential diagnosis of the disease, mycosis ^12^ and Chagas disease ^15^.

On the other hand, serum samples from pregnant patients, immunosuppressed patients or hemolyzed samples or those with signs of contamination were excluded.

### Statistical analysis

We determined frequency measures and percentages, as well as contingency tables with the obtained results. The diagnostic performance of *Leishmania (V.) braziliensis* and *Leishmania (V.) peruviana* antigens was assessed against the DME reference method for the cutaneous type and the IIF method for the mucosal form, using the X^2^ distribution with Bonferroni adjustment.

Estimators and 95% confidence intervals were calculated for diagnostic performance measures such as sensitivity, specificity ^16^, area under the ROC curve, positive predictive value, negative predictive value, positive likelihood ratio, negative likelihood ratio for each of the antigens. The statistical program Stata v17.1 (Stata Corporation, College Station, Texas, USA) was used for the analysis using a significance level of 0.05.

## RESULTS

In tour study, 87.2% of the samples corresponded to patients with DME-confirmed ATL and 12.8% were from patients without suspected disease (Supplementary Material Table S1).

SDS-PAGE electrophoresis showed that soluble and ES antigens presented a protein profile from approximately 15 kDa to 250 kDa for both *Leishmania braziliensis* and *Leishmania peruviana* (Supplementary Material Figure S1).

The evaluation against the control sera showed that the soluble antigens of *Leishmania (V. ) braziliensis* had a sensitivity of 87.7% (95%CI: 80.8-92.8) and specificity of 100% (95%CI: 85.2-100), on the other hand, among the groups, it had a sensitivity of 96% (IC95%: 90.1-98.9) for the cutaneous form and 60% (IC95%: 40.6-77.3) for the mucosal type; it also presented cross-reactivity, 20% against sera from patients with Chagas disease and 8.3% against patients with mycosis.

The soluble *Leishmania (V.) peruviana* antigen had a sensitivity of 92.3% (95%CI: 86.3-96.2) and specificity of 95.7%; between groups it had a sensitivity of 91% for the cutaneous form, 97.7% for the mucosal type, as detailed in Table 1, and a cross-reactivity of 33.3% and 50% for Chagas disease and mycosis, respectively (Table 2).

**Table 1:** Diagnostic performance of soluble and excretion/secretion Ag. of *Leishmania braziliensis* and *Leishmania peruviana*.

		LbSA (95%CI)	LpSA (95%CI)	LbE/S (95%CI)	LpE/S (95%CI)
Tegumentary leishmaniasis (Cutaneous and mucosal)	Sensitivity	87.7 (80.8; 92.8)	92.3 (86.3; 96.2)	16.9 (10.9; 24.5)	43.8 (35.2; 52.8)
	Specificity	100 (85.2; 100)	95.7 (78.1; 99.9)	100 (85.2; 100)	82.6 (61.2; 95.0)
	Area under the ROC curve	0.954 (0.91; 0.97)	0.94 (0.89; 0.99)	0.58 (0.55; 0.62)	0.63 (0.54; 0.72)
	Positive predictive value	100 (96.8; 100)	99.2 (95.5; 100)	100 (84.6; 100)	93.4 (84.1; 98.2)
	Negative predictive value	59.0 (42.1; 74.4)	68.8 (50.0; 83.9)	17.6 (11.5; 25.2)	20.7 (12.9; 30.4)
	Positive likelihood ratio	41.95 (2.7; 652.06)	21.23 (3.12; 144.46)	8.24 (0.52; 131.36)	2.52 (1.01; 6.27)
	Negative likelihood ratio	0.13 (0.08; 0.2)	0.08 (0.04; 0.15)	0.85 (0.77; 0.93)	0.68 (0.53; 0.87)
Cutaneous leishmaniasis	Sensitivity	96.0 (90.1; 98.9)	91.0 (83.6; 95.8)	1.0 (0.0; 5.4)	35.0 (25.7; 45.2)
	Specificity	100 (85.2; 100)	95.7 (78.1; 99.9)	100 (85.2; 100)	82.6 (61.2; 95.0)
	Area under the ROC curve	0.98 (0.96; 1.00)	0.93 (0.88; 0.98)	0.50 (0.50; 0.51)	0.59 (0.50; 0.68)
	Positive predictive value	100 (96.2; 100)	98.9 (94.1; 100)	100 (2.5; 100)	89.7 (75.8; 97.1)
	Negative predictive value	85.2 (66.3; 95.8)	71.0 (52.0; 85.8)	18.9 (12.3; 26.9)	22.6 (14.2; 33.0)
	Positive likelihood ratio	45.86 (2.95; 712.46)	20.93 (3.08; 142.46)	0.71 (0.03; 16.96)	2.01 (0.79; 5.10)
	Negative likelihood ratio	0.05 (0.02; 0.11)	0.09 (0.05; 0.18)	1.01 (0.94; 1.07)	0.79 (0.62; 1.00)
*Leishmaniasis mucosa*	Sensitivity	60.0 (40.6; 77.3)	96.7 (82.8; 99.9)	70.0 (50.6; 85.3)	73.3 (54.1; 87.7)
	Specificity	100 (85.2; 100)	95.7 (78.1; 99.9)	100 (85.2; 100)	82.6 (61.2; 95.0)
	Area under the ROC curve	0.80 (0.71; 0.89)	0.96 (0.91; 1.00)	0.85 (0.77; 0.93)	0.78 (0.67; 0.89)
	Positive predictive value	100 (81.5; 100)	96.7 (82.8; 99.9)	100 (83.9; 100)	84.6 (65.1; 95.6)
	Negative predictive value	65.7 (47.8; 80.9)	95.7 (78.1; 99.9)	71.9 (53.3; 86.3)	70.4 (49.8; 86.2)
	Positive likelihood ratio	28.65 (1.82; 451.69)	22.23 (3.27; 151.35)	33.29 (2.12; 522.18)	4.22 (1.69; 10.54)
	Negative likelihood ratio	0.41 (0.27; 0.63)	0.03 (0.01; 0.24)	0.31 (0.18; 0.53)	0.32 (0.17; 0.60)

95%CI=95% confidence interval. LbSA: Leishmania braziliensis soluble antigen; LpSA: Leishmania peruviana soluble antigen; LbE/S: Leishmania braziliensis expression/secretion antigen; LpE/S: Leishmania peruviana expression/secretion antigen.

**Table 2:** Cross-reactivity of soluble antigens and ES antigens of *Leishmania braziliensis* and *Leishmania peruviana*.

		Result		S (95%CI)	Cross-reactivity
		TN	FP		FPP (FP/n)
Chagas disease (n=15)					
	LbSA	12	3	80.0 (51.9; 95.7)	20.0
	LpSA	10	5	66.7 (38.4; 88.2)	33.3
	LbE/S	15	0	100 (78.2; 100)	0.0
	LpE/S	15	0	100 (78.2; 100)	0.0
Mycosis (n=12)					
	LbSA	11	1	91.7 (61.5; 99.8)	8.3
	LpSA	6	6	50.0 (21.1; 78.9)	50.0
	LbE/S	12	0	100 (73.5; 100)	0.0
	LpE/S	12	0	100 (73.5; 100)	0.0
Overall (n=27)					
	LbSA	23	4	85.2 (66.3; 95.8)	14.8
	LpSA	16	11	59.3 (38.8; 77.6)	40.7
	LbE/S	27	0	100 (87.2; 100)	0.0
	LpE/S	27	0	100 (87.2; 100)	0.0

S: specificity; 95%CI=95% confidence interval. LbSA: *Leishmania braziliensis* soluble antigen; LpSA: *Leishmania peruviana* soluble antigen; LbE/S: *Leishmania braziliensis* excretion/secretion antigen; LpE/S: *Leishmania peruviana* excretion/secretion antigen; TN: true negatives; FP: false positives; FPP: false positive proportion.

**Figure 1:**
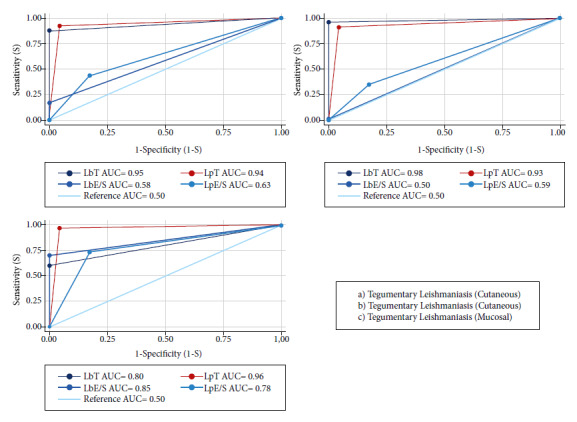
Comparison of the area under the curve of soluble and excretion/secretion antigens of *Leishmania (V.) braziliensis* and *Leishmania (V.) peruviana*. Where LbT: soluble antigen of *Leishmania braziliensis*; LpT: soluble antigen of *Leishmania peruviana*; LbE/S: excretion/secretion antigen of *Leishmania braziliensis*: LpE/S: excretion/secretion antigen of *Leishmania peruviana*: AUC: area under the curve.

Likewise, the evaluation of concordance in the detection of ATL showed, considering the fractions between 50-55 kDa as positivity criteria (Figure 1), that the soluble antigen of *Leishmania (V.) braziliensis* had a kappa index of 0.68 and 0.76 for *Leishmania (V.) peruviana*; on the other hand, among the cutaneous and mucosal groups, the soluble antigen of *Leishmania (V.) braziliensis* had a kappa index of 0.9 and 0.76, respectively, and in case of *Leishmania (V.) peruviana*, 0.56 for the cutaneous and 0.92 for the mucosal form of the disease (Supplementary Material Table S2).

Regarding the diagnostic performance of ATL, the LbSA antigen had an area under the curve of 0.95; it was 0.98 for the cutaneous form and 0.80 for the mucosal; while, the LpSA reached an area under the curve of 0.94 in the detection of ATL; 0.93 for the cutaneous form and 0.96 for the mucosal, as detailed in Table 1 and Supplementary Material Figure S3. 

## DISCUSSION

In this study, the immunoblot method has allowed, among other things, to demonstrate the vast diversity of proteins presented by *Leishmania* spp ^17^^,^^18^, the antigen-antibody interaction by means of an enzyme-linked immunosorbent reaction, and the identification of potential biomarkers that could improve the serological diagnosis of the disease ^19^.

Related studies, using antigen obtained from *Leishmania major* MRHO/IR/75/ER, determined that the protein with the best performance in the detection of anti-Leishmania antibodies was 63 kDa, this fraction had a sensitivity of 96.7% and a specificity of 70% ^20^, whereas, Brito *et al*, (2000) using *Leishmania braziliensis* MHOM/BR/75/M2903 proteins against sera from confirmed patients, reported as immunogenic proteins those of molecular weight 30 and 27 kDa, which were recognized in 88% and 91% of the cases, the 48 kDa protein fraction in 70%, and those of 60 kDa and 66 kDa in less than 35% of the samples. Considering the visualization of these protein fractions as a criterion for positivity in the detection of anti-Leishmania antibodies, they reported a sensitivity of 91% and specificity of 100% ^21^; and 76.9% and 100%, respectively, using *Leishmania (V.) braziliensis* MHOM/BR/1987/M11272 antigens, considering proteins of molecular weight 42, 58 and 63 kDa as criteria for positivity ^17^.

In our study, the immunogenic proteins with the best performance in the detection of ATL were between 50-55 kDa, which reached a sensitivity of 87.7% and specificity of 100% using *Leishmania (V.) braziliensis*, and 92.3% and 95.7%, respectively, in *Leishmania (V.) peruviana*. In contrast, the 63, 42, 30 and 27 kDa proteins previously reported showed a frequency of less than 15% against cutaneous and mucosal sera in the samples included in the study.

These differences in the immunogenic character of *Leishmania* are probably related to the intrinsic characteristics of each species and even within the same species, although previous studies used the strains of *Leishmania braziliensis* MHOM/BR/75/M2903 and MHOM/BR/1987/M11272, both correspond to isolates from Brazil, on the contrary, this study was developed using the strain MHOM/PE/84/LC53 isolated in Peru. It should be pointed out that studies on *Leishmania braziliensis* have demonstrated genetic divergence even in those isolated within the same geographical area ^22^, each of them with clearly differentiable characteristics thanks to current methodologies. For this reason, the immunogenic character found in this study could be related to the genetic component of the species.

ES antigens showed high specificity, but limited sensitivity, a result that resembles that reported by Longoni *et al*. (2014), who, from strains of *Leishmania amazonensis* and *Leishmania peruviana* and employing soluble antigens, reported a sensitivity of 21.6% and 56.9% respectively, and when using iron superoxide dismutase secreted antigens in both species the sensitivity was 82.4% and 11.8%, respectively ^23^.

Currently, indirect immunofluorescence is a widely used method for the serological diagnosis of the disease, it has variable sensitivity and specificity which depends on factors such as the time of evolution of the disease and the clinical form of the disease, in addition to the species of *Leishmania* used as antigen, in that sense, using promastigotes of *Leishmania infantum* and sera of immunosuppressed patients with visceral leishmaniasis, the IIF method presented a sensitivity of 79.4% and specificity of 99.2% ^8^. On the other hand, for the tegumentary form, the sensitivity of the method in the detection of ATL was 90% and specificity between 62% and 100% ^8^^,^^19^.

The cross-reactivity assessed against sera from patients with paracoccidioidomycosis, toxoplasmosis and Chagas disease, using *Leishmania amazonensis*, was 23.4%, 0% and 70%, respectively, and it was 23.4%, 12.5% and 80% using *Leishmania braziliensis* promastigotes, respectively ^24^. In Peru, considering samples from patients confirmed for the disease with a disease length of more than 2 months and using *Leishmania braziliensis* promastigotes as antigen, the National Referral Laboratory of Metaxenics and Parasitic Zoonoses reports a sensitivity of 83.95% (95%CI 75.34 - 92.56), specificity of 82.76% (95%CI: 72.2-93.3) and cross-reactivity mainly with Chagas disease (unpublished data).

Our study has some limitations, such as the low number of mucosal form samples, the limited number of samples from other diseases, as well as the absence of negative control coming from an endemic area. Therefore, we were not able to identify the protein fractions that interact in this group of samples, which could represent bias, so we recommend continuing the research of these antigens using a greater number of samples.

In conclusion, our study showed that the proteins of approximately 50-52 and 55 kDa of the soluble antigen of *Leishmania braziliensis* had a sensitivity of 88% and specificity of 100%, while the sensitivity and specificity found from the soluble antigens of *Leishmania peruviana* were 92% and 96%, respectively.

According to our findings, it is important to develop validation of the immunoblot method using soluble antigens of *Leishmania braziliensis* and *Leishmania peruviana*, and we recommend the development of studies aimed at the characterization and analysis of the reported immunogenic regions in order to continue with the development of recombinant and synthetic proteins, aimed at improving the efficiency of the serological diagnosis of the disease.
