# Supplementary material for: Diagnostic performance of Leishmania braziliensis and Leishmania peruviana antigens in the immunoblot method for the detection of american tegumentary leishmaniasis
Source: Rev Peru Med Exp Salud Publica. 2024 Aug 28;41(3):294–300. doi: 10.17843/rpmesp.2024.413.13231 (PMC11495926; doi:10.17843/rpmesp.2024.413.13231)
Supplement: Supplementary material. — Available in the electronic version of the RPMESP. [file rpmesp-41-03-13231-s001.docx]

Material suplementario

**S1. Metodología**

Antígenos empleados

El antígeno soluble (AS) y de excreción/secreción (AES) fueron obtenidos de promastigotes de *Leishmania braziliensis* y *Leishmania peruviana.* Las cepas criopreservados en glicerol a -80°C fueron descongeladas e inmediatamente inoculadas en medio bifásico agar sangre y medio liquido Drosophila de Schneider (Gibco–Life technologies) suplementado con 10% de suero fetal bovino y gentamicina (medio Schneider completo) e incubados a 26°C, se adicionó medio Schneider completo en la proporción aproximadamente v/v cada 24 horas hasta el quinto día post inoculación.

Obtención del Ag soluble.

Los promastigotes en fase exponencial, fueron centrifugados a 8000 rpm x 10 minutos, se descartó cuidadosamente el sobrenadante y el sedimento resuspendido con solución salina fisiológica 0.89% (SSF), luego centrifugado nuevamente a 8000 rpm x 10 minutos, este proceso fue repetido dos veces.

El sedimento fue resuspendido con SSF, luego sonicado en cadena de frio por 5 ciclos, cada ciclo por 1 minuto de sonicado y 1 minuto de reposo, al término, fue centrifugado a 10 000 rpm x 5 minutos y el sobrenadante cuidadosamente recuperado. La concentración proteica fue determinada mediante el método de Bradford (Bradfrod Reagent, Sigma). Al término, se añadió inhibidor de proteasas y conservó a congelación a -40°C hasta su uso.

Obtención de los antígenos ES.

Promastigotes en fase exponencial en 200 ml de medio a una concentración de 10^7^ parásitos/ml fueron centrifugados a 4000 rpm x 10 minutos, se descartó el sobrenadante y el sedimento resuspendido con SSF, este proceso fue repetido 2 veces.

El sedimento fue resuspendido con SSF hasta un volumen de 200 ml, luego incubado a 26°C por 48 horas, la viabilidad de los parásitos fue monitorizado en microscopio invertido.

La recuperación antígenos ES se realizó por centrifugación a 4000 rpm x 10 minutos, se cuantificó y conservó de manera similar a las proteínas del Ag soluble.

Electroforesis de proteínas.

Se realizó en condiciones denaturantes SDS-PAGE en el equipo Mini-PROTEAN® Tetra Cell (BioRad), se empleó la solución de acrilamida al 30% (14.6 gr acrilamida, 0.4 gr N’N’-bis metileno acrilamida en un volumen final de 50 ml).

Se preparó gel de acrilamida al 10% (4.4 mL solución de acrilamida; 3.3 mL Tris aminometano 1.5 M pH 8.8; 0.12 mL SDS al 10%; 4.3 mL ddH_2_O; 60 µL APS al 10%; 4 µL Temed) para la separación de proteínas y gel de acrilamida 4% como empaquetamiento (1.33 mL solución de acrilamida; 2.5 mL Tris HCl 0.5 M pH 6.8; 0.1 mL SDS al 10%; 6.1 mL ddH_2_O; 50 µL persulfato de amonio APS al 10%; 5 µL Temed).

Las proteínas del antígenos solubles y de ES de *Leishmania braziliensis* (LbAS y LbAES) y de *Leishmania peruviana* (LpAS y LpAES) fueron tratadas con la solución 0.5 M Tris-HCl pH 6.8, 0.5% β-mercaptoetanol, 20 mg SDS a la proporción 1:1, se sometió a ebullición por 5 minutos, se añadió al volumen final 3.3% de solución colorante de corrida (50 mg azul de bromofenol, 80% glicerol, 0.5 M Tris-HCl pH 8.0, 1% ddH_2_O).

La electroforesis fue realizada a 15 mA/gel hasta alcanzar el gel de separación donde se incrementó a 30 mA por gel por 60 minutos.

Al término, se retiró cuidadosamente el gel, se descartó el gel de empaquetamiento, mientras que, el gel de separación fue llevado a tinción con plata para la visualización del desplazamiento de las proteínas.

Transferencia de proteínas en membranas de nitrocelulosa

Se realizó SDS-PAGE al 10% de poliacrilamida, se empleó el molde Prep+1de 67 mm para carga de muestra, se empleó los antígenos a la concentración de 4 µg/mm y una solución conteniendo proteínas de peso molecular conocido entre 6 – 250 kDa (SeeBlue® Plus2 Pre-Stained Standard, BioRad) como marcador estándar.

La transferencia de proteínas, se realizó en gel de poliacrilamida sobre una membrana de nitrocelulosa de 0.22 µm embebido en la solución tampón Towbin pH 9.2 (0.025 M de Tris aminometano, 0.192 M de glicina y 20% de metanol) este proceso se realizó a 55 voltios por 1 hora.

Al término, se recuperó cuidadosamente la membrana de nitrocelulosa, y con la finalidad de verificar el proceso de trasferencia de proteínas a la membrana de nitrocelulosa, un extremo de la membrana fue teñido con tinta china diluido al 1%.

La membrana de nitrocelulosa fue sometido a cinco lavados con solución fosfato salino (PBS) 0.01 M pH 7.2 con Tween 20 al 0.3% (PBS-T20 o solución de lavado), se realizó un lavado adicional solo con PBS. Se bloqueó la membrana con PBS-T20 y leche descremada al 5% (solución de bloqueo/diluyente de muestras) y se llevó a incubación a temperatura ambiente con agitación de 100 rpm por 30 minutos.

Al término, se secó la membrana a temperatura ambiente entre 30 a 60 minutos aproximadamente, luego se procedió a cortar tiras de 4 mm, dispuestos en tubos de vidrio 13 x 100 y conservados en refrigeración hasta el momento de su uso.

Reacción enzimática

Para las tiras de nitrocelulosa conteniendo antígenos solubles, se empleó sueros diluidos a la proporción de 1:200, mientras que, para el antígenos ES a 1:100, las muestras fueron incubadas por 1 hora en agitación a 100 rpm. Se realizó 3 lavados con 1 ml de PBS-T20 y agitación por 10 minutos.

Se adicionó el anticuerpo secundario Anti IgG humano ligado a peroxidasa (Sigma Aldrich) diluido a la proporción 1:3000 para antígenos solubles y 1:2000 para antígenos ES, las muestras fueron incubadas a temperatura ambiente por 1 hora en agitación constante, al término, se realizó nuevamente 3 lavados y un lavado con PBS por 10 minutos a 100 rpm.

La reacción enzimática fue visualizada por adición de diaminobenzidina y peróxido de oxígeno e incubada a temperatura ambiente por 5 a 10 minutos en agitación, la reacción se detuvo mediante lavados consecutivos con agua destilada.

**Determinación de la sensibilidad, especificidad y reactividad cruzada**.

La sensibilidad fue determinada del procesamiento de las 130 muestras de suero de LTA, estuvo conformado por 100 muestras de suero de la forma cutánea confirmados por EMD y 30 sueros de la forma mucosa confirmado por la ficha clínica epidemiológica además del resultado positivo en el método de IFI.

Mientras que, la especificidad fue obtenida empleando 30 sueros de pacientes sanos sin sospecha clínica de la enfermedad y negativos a IFI.

Por otro lado, la reactividad cruzada se obtuvo de la evaluación de 27 muestras de suero de pacientes de otras patologías involucrados en el diagnóstico diferencial de la enfermedad, micosis y la enfermedad de Chagas.

Tabla S1. Frecuencia de las muestras incluidas en el estudio.

|  | **n / N** | **%** |
| --- | --- | --- |
| **Tipo de muestra** |  |  |
| Sin infección | 23 / 180 | 12,8 |
| Con infección | 157 / 180 | 87,2 |
| **Tipo de infección** |  |  |
| Leishmaniasis tegumentaria americana | 130 / 157 | 82,8 |
| Cutánea | 100 / 130 | 76,9 |
| Mucosa | 30 / 130 | 23,1 |
| Otra patología | 27 / 157 | 17,2 |
| Enfermedad de Chagas | 15 / 27 | 55,6 |
| Micosis | 12 / 27 | 44,4 |
|  |  |  |
| n=Cantidad de muestras en la categoría, N=Cantidad total de muestras para el grupo, %=Porcentaje.  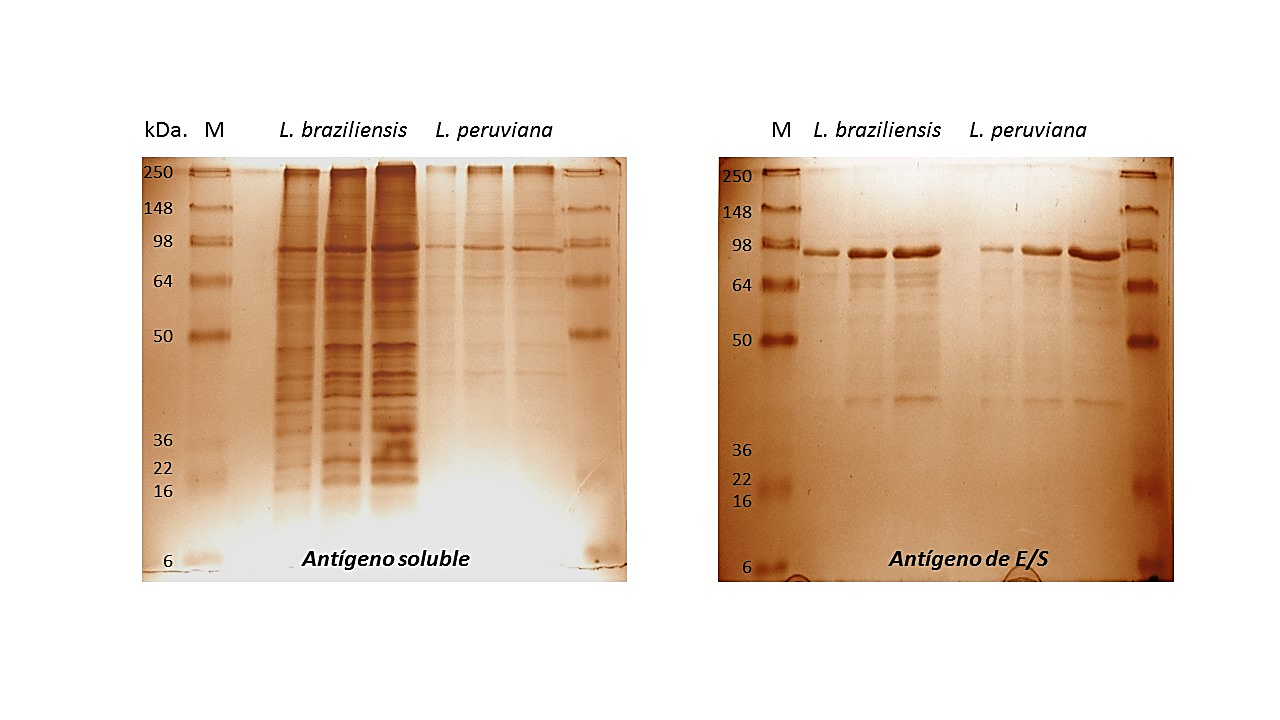  **Figura S1.** Perfil electroforesis de proteínas de los antígenos solubles y E/S de Leishmania braziliensis y Leishmania peruviana. M. Marcador estándar (kDa).  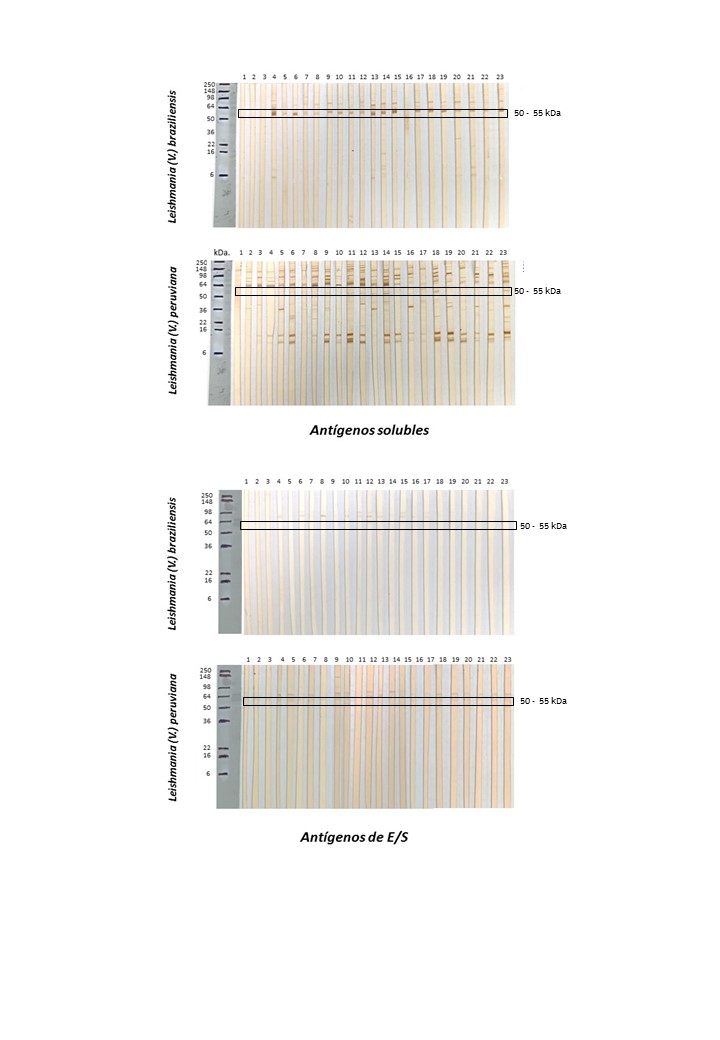**Figura S2.** Reacción enzimática de los antígenos solubles y E/S de *Leishmania (V.) braziliensis* y *Leishmania (V.) peruviana* frente a muestras de pacientes confirmados para la enfermedad. El recuadro corresponde a las proteinas entre 50 a 55 kDa. | | |

Tabla S2. Grado de acuerdo obtenido de los antígenos solubles y de E/S de Leishmania braziliensis y Leishmania peruviana con respecto a la presencia de la enfermedad.

|  |  | **LTA (Cutánea y mucosa)** | | **Kappa**  **(IC 95%)** | ***Valor de P*** | **Grado de Acuerdo** |  | **LTA (Cutánea)** | | **Kappa**  **(IC 95%)** | **Valor de P** | **Grado de acuerdo** |  | **LTA (Mucosa)** | | **Kappa**  **(IC 95%)** | ***Valor de P*** | **Grado de acuerdo** |  |
| --- | --- | --- | --- | --- | --- | --- | --- | --- | --- | --- | --- | --- | --- | --- | --- | --- | --- | --- | --- |
|  |  | **Con infección** | **Sin Infección** |  |  |  |  | **Con infección** | **Sin Infección** |  |  |  |  | **Con infección** | **Sin Infección** |  |  |  |  |
| **Ag. Soluble** | L. braziliensis |  |  |  |  |  |  |  |  |  |  |  |  |  |  |  |  |  |  |
|  | Positivo | 114 | 0 | 0,682  (0,542; 0,822) | <0,001 | Sustancial |  | 96 | 0 | 0,900  (0,804; 0,996) | <0,001 | Casi perfecto |  | 18 | 0 | 0,566  (0,371; 0,760) | <0,001 | Moderado |  |
|  | Negativo | 16 | 23 |  |  |  |  | 4 | 23 |  |  |  |  | 12 | 23 |  |  |  |  |
|  | L. peruviana |  |  |  |  |  |  |  |  |  |  |  |  |  |  |  |  |  |  |
|  | Positivo | 120 | 1 | 0,758  (0,623; 0,892) | <0,001 | Sustancial |  | 91 | 1 | 0,764  (0,627; 0,901) | <0,001 | Sustancial |  | 29 | 1 | 0,923  (0,819; 1,000) | <0,001 | Casi perfecto |  |
|  | Negativo | 10 | 22 |  |  |  |  | 9 | 22 |  |  |  |  | 1 | 22 |  |  |  |  |
| **Ag. E/S** | L. braziliensis |  |  |  |  |  |  |  |  |  |  |  |  |  |  |  |  |  |  |
|  | Positivo | 22 | 0 | 0,058  (0,025; 0,090) | 0,017 | Insignificante |  | 1 | 0 | 0,004  (-0,004; 0,011) | 0,315 | Insignificante |  | 21 | 0 | 0,669  (0,484; 0,855) | <0,001 | Insignificante |  |
|  | Negativo | 108 | 23 |  |  |  |  | 99 | 23 |  |  |  |  | 9 | 23 |  |  |  |  |
|  | L. peruviana |  |  |  |  |  |  |  |  |  |  |  |  |  |  |  |  |  |  |
|  | Positivo | 57 | 4 | 0,118  (0,028; 0,208) | 0,009 |  |  | 35 | 4 | 0,087  (-0,008; 0,182) | 0,051 | Insignificante |  | 22 | 4 | 0,548  (0,326; 0,771) | <0,001 | Moderado |  |
|  | Negativo | 73 | 19 |  |  | Insignificante |  | 65 | 19 |  |  |  |  | 8 | 19 |  |  |  |  |
|  | Total | 130 | 23 |  |  |  |  | 100 | 23 |  |  |  |  | 30 | 23 |  |  |  |  |
|  |  |  |  |  |  |  |  |  |  |  |  |  |  |  |  |  |  |  |  |
| **IC 95%= Intervalo de confianza al 95%.** | | | | | | | | | | | | | | | | | |  |  |


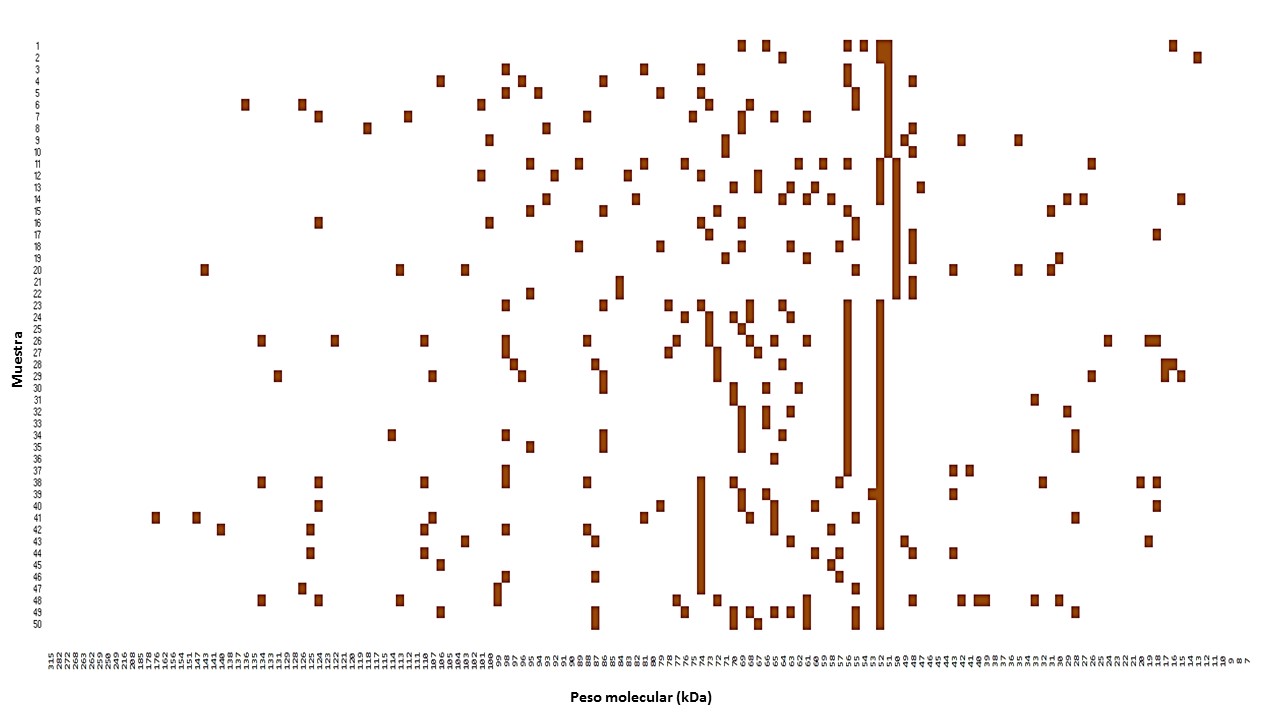


**Muestra**


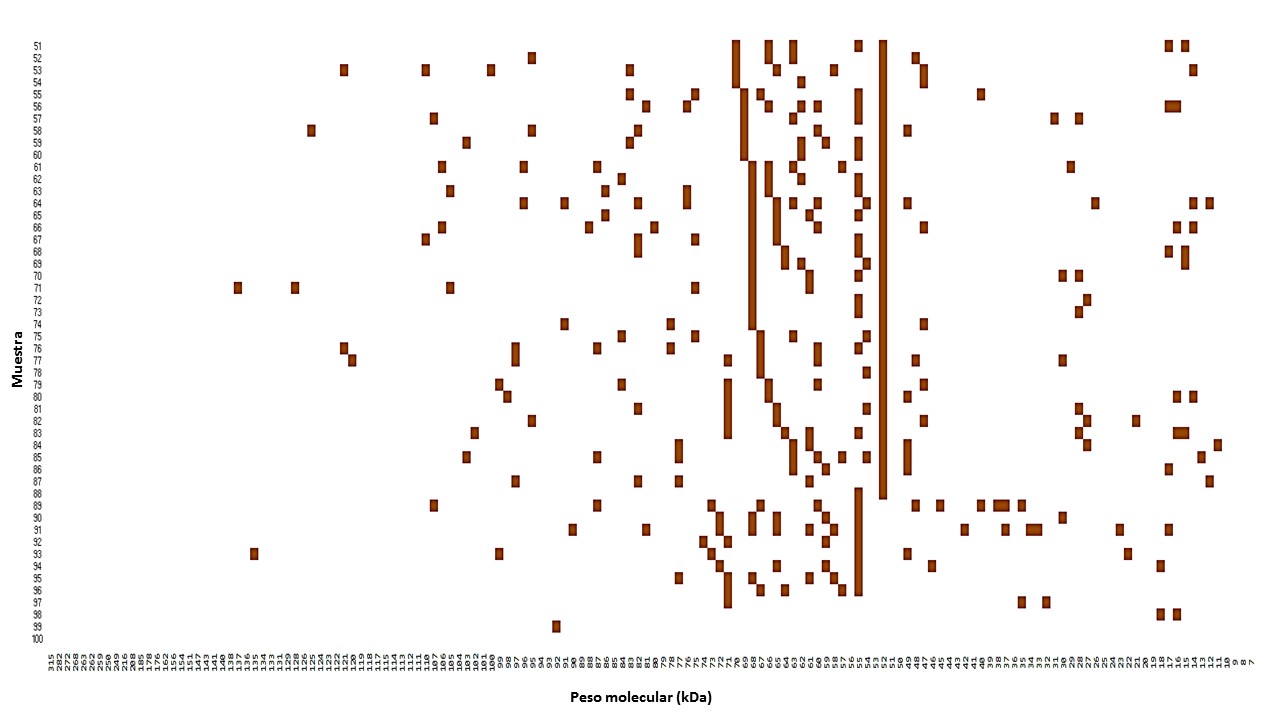


Figura S3. Reacción antígeno - anticuerpo empleando los antígenos solubles de Leishmania (Viannia) braziliensis frente a sueros de pacientes con leishmaniasis cutánea.


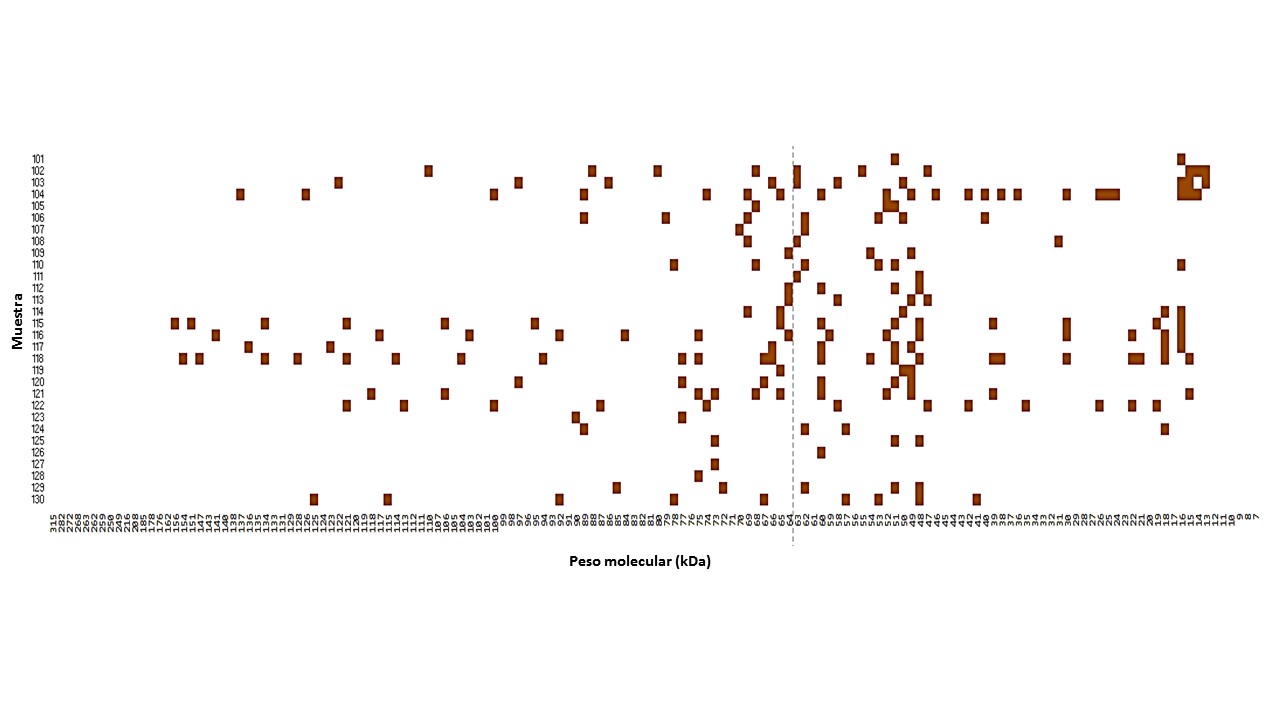


Figura S4. Reacción antígeno - anticuerpo empleando los antígenos solubles de Leishmania (Viannia) braziliensis frente a sueros de pacientes con leishmaniasis mucosa.

*
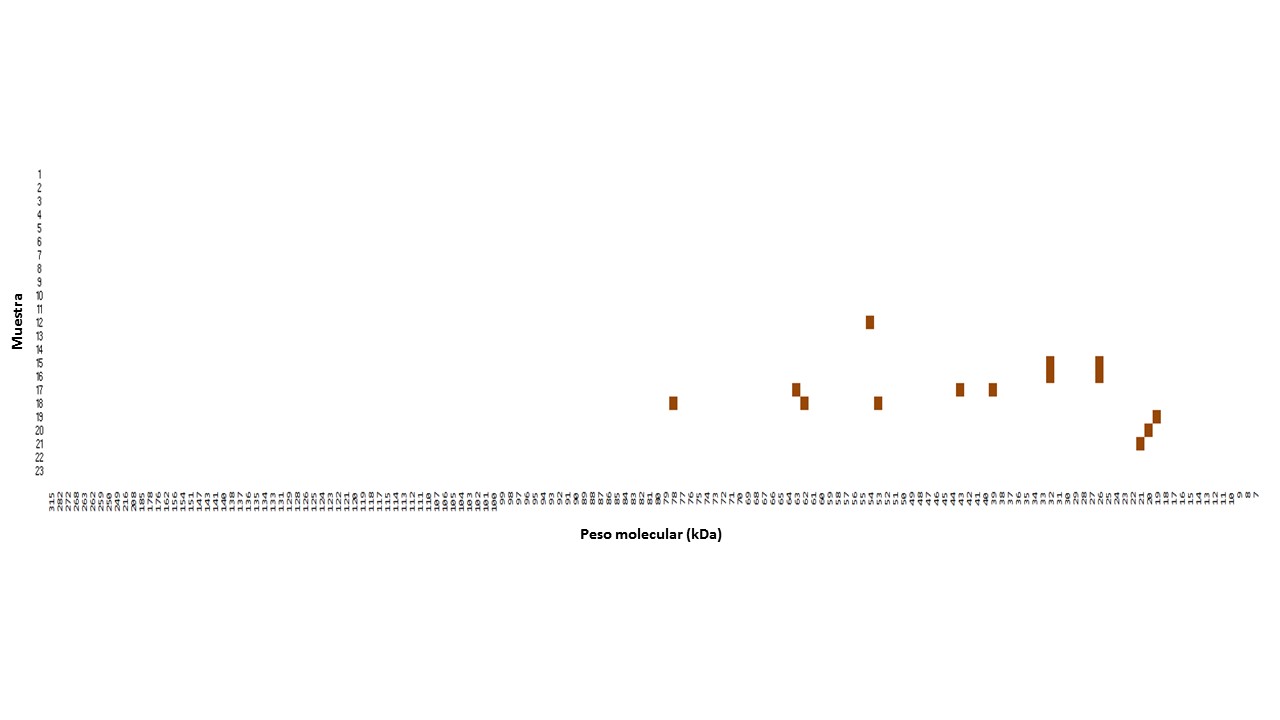
*

Figura S5. Reacción antígeno - anticuerpo empleando los antígenos solubles de *Leishmania (Viannia) braziliensis* frente a sueros del grupo control.
