# Supplementary material for: Diagnostic performance of Leishmania braziliensis and Leishmania peruviana antigens in the immunoblot method for the detection of american tegumentary leishmaniasis
Source: Rev Peru Med Exp Salud Publica. 2024 Aug 28;41(3):294–300. doi: 10.17843/rpmesp.2024.413.13231 (PMC11495926; doi:10.17843/rpmesp.2024.413.13231)
Supplement: Translation

# Translation: Rendimiento diagnóstico de los antígenos de *Leishmania braziliensis* y *Leishmania peruviana* en el método de inmunoblot para la detección de la leishmaniasis tegumentaria americana

**Authors:** Nyshon Rojas-Palomino, Aidé Sandoval-Juarez, Gilmer Solis-Sánchez, Gloria Minaya-Gómez

**Affiliations:** 1 Centro Nacional de Salud Pública, Instituto Nacional de Salud, Lima, Perú; 2 Centro Nacional de Alimentación, Nutrición y Vida Saludable, Instituto Nacional de Salud, Lima, Perú.

**Keywords:** Leishmaniasis, Leishmania, Western Blotting, Leishmania braziliensis, Antígenos, Sensibilidad y Especificidad

## Abstract

El presente estudio se planteó determinar el rendimiento de antígenos de *Leishmania braziliensis* y *Leishmania peruviana* en la detección de LTA, fue desarrollado a partir de muestras de suero obtenidas entre 2013 - 2016. Los antígenos solubles y de excreción/secreción obtenidos fueron transferidos a membrana de nitrocelulosa mediante un ensayo de inmunotransferencia. Se realizó la evaluación frente a sueros confirmados para LTA, a un nivel de confianza al 95%, logrando determinar que, el antígeno soluble de *Leishmania braziliensis* presenta una sensibilidad del 87,7%, especificidad del 100% y área bajo la curva de 0,95; mientras que, *Leishmania peruviana* se encontró valores de 92,3%, 95,7% y 0,94 respectivamente. De acuerdo a los resultados, recomendamos realizar la caracterización y análisis de las regiones inmunogénicas reportadas a fin de continuar con el desarrollo de proteínas recombinantes y sintéticas, orientadas a mejorar la eficiencia del diagnóstico serológico de la enfermedad.

## INTRODUCCIÓN

La Leishmaniasis tegumentaria americana (LTA), enfermedad causada por más de 20 especies de *Leishmania* agrupa un conjunto de manifestaciones clínicas desde lesiones únicas hasta múltiples, nodulares, en placas, entre otros, hasta lesiones que pueden comprometer mucosas. De acuerdo con la Organización Mundial de la Salud (OMS), nuestro continente reporta más de 1 millón de casos, siendo Brasil, Colombia y Perú los países que en conjunto registran el 72% del total de casos de LTA ^1^^,^^2^.

En Perú, la LTA es causada por 8 especies siendo *Leishmania braziliensis* de mayor importancia debido al daño que puede infligir en el paciente, desde la forma cutánea localizada y diseminada hasta la mucosa. Otras especies reportadas son *Leishmania peruviana, Leishmania guyanensis, Leishmania panamensis, Leishmania shawi, Leishmania lainsoni, Leishmania naiffi* y *Leishmania amazonensis*^3^*.* Esta enfermedad se reporta en 19 regiones del país, donde se ha registrado más de 156 mil casos, de los cuales aproximadamente 10 mil (6,4%) corresponde a la forma mucosa ^3^^,^^4^.

El diagnóstico se realiza principalmente mediante métodos parasitológicos como el Examen Microscópico Directo (EMD) con tinción Giemsa y cultivo *in vitro*, ambos confirman la enfermedad por visualización microscópica del parásito, estos métodos presentan elevada especificidad, pero limitada sensibilidad debido principalmente a la forma clínica y el tiempo de evolución de la enfermedad, no es recomendado en pacientes que presenten lesiones cutáneas crónicas o manifiesten compromiso mucoso, por el escaso número de amastigotes que presenta la lesión, que dificulta su detección ^5^^,^^6^. Otros factores son la infección bacteriana sobreagregada, la falta de experiencia en la obtención de la muestra y el reconocimiento de las formas amastigotes ^5^^,^^7^.

Los métodos serológicos como Inmunofluorescencia Indirecta (IFI) y ELISA, permiten el diagnóstico por detección de anticuerpos anti-*Leishmania*, principalmente en pacientes con lesiones cutáneas crónicas con sospecha clínica y en aquellos que presentan compromiso mucoso ^8^. En Perú, el IFI con parásito completo como antígeno es ampliamente empleado en el diagnóstico, a pesar de las limitaciones asociadas con la reactividad cruzada principalmente frente a la enfermedad de Chagas debido a la similitud genómica y proteómica considerable entre *Leishmania* spp y *Trypanosoma cruzi*^9^^*,*^^10^*.*

Asimismo, la eficiencia de los métodos serológicos está ligado a la naturaleza del antígeno, estructura y ubicación de la proteína empleada, procedencia del antígeno, especie o estadio del cual fue obtenido, entre otros, la miscelánea proteínica producto de la expresión génica del parásito afecta el rendimiento diagnóstico del método ^11^^,^^12^.

Por tanto, actualmente se vienen desarrollando estudios relacionados a la identificación y evaluación de biomarcadores serológicos orientados a mejorar la eficiencia del método ^13^^,^^14^, reduciendo la variabilidad inter e intra especie e inclusive de la respuesta inmunológica por la especie infectante ^15^.

En ese sentido, nuestro objetivo fue evaluar y determinar el rendimiento diagnóstico de los antígenos solubles y de excreción/secreción de *Leishmania braziliensis* y *Leishmania peruviana* en el Inmunoblot frente a sueros de pacientes confirmados para la detección de la Leishmaniasis tegumentaria americana.

**MENSAJES CLAVE**

Motivación para realizar el estudio. Aportar sobre el carácter inmunogénico de los antígenos solubles y de excreción/secreción de *Leishmania braziliensis* y *Leishmania peruviana* con la finalidad de identificar proteínas con potencial diagnóstico.

Principales hallazgos. El antígeno soluble de *Leishmania braziliensis* presenta una sensibilidad en la detección de LTA del 87,7%, especificidad del 100% y una proporción de falsos positivos de 20% frente a sueros de pacientes con la enfermedad de Chagas y del 8,3% con micosis.

Implicancias. El Inmunoblot puede mejorar la capacidad resolutiva en el diagnóstico serológico de Leishmaniasis tegumentaria americana, especialmente en pacientes donde el tiempo de la enfermedad y la forma clínica hacen difícil el diagnóstico mediante los métodos parasitológicos.

## EL ESTUDIO

### Diseño del estudio

Se realizó un estudio observacional de pruebas diagnósticas en el Laboratorio de Referencia Nacional de Metaxénicas y Zoonosis Parasitaria (LRN MEZOP) del Instituto Nacional de Salud entre 2013 - 2016, aprobado por el Comité de Investigación y Ética del Instituto Nacional de Salud con RD N°170-2013-DG-OGITT-OPE/INS.

### Población y muestra

La población de estudio estuvo conformada por sueros almacenados en la seroteca del LRN MEZOP-INS obtenidos en marco de las actividades de diagnóstico especializado de LTA entre el año 2013-2016.

El tamaño de la muestra fue determinado empleando el programa Epidat v3.0 considerando una especificidad esperada del 98%, 5% de precisión, proporción de sanos/enfermos de 0,25; a un nivel de confianza del 95%.

Se seleccionó de manera aleatoria un total de 187 muestras de suero considerando el método del EMD como criterio de elegibilidad para la forma cutánea y el antecedente clínico epidemiológico además del resultado en IFI para la forma mucosa.

Estuvo conformada por 100 sueros de la forma cutanea y 30 de la forma mucosa, confirmados por visualización microscópica de las formas amastigotes en EMD o por inmunofluorescencia indirecta más el antecedente clínico epidemiológico respectivamente; 30 sueros de pacientes sanos sin sospecha clínica de la enfermedad procedentes de área no endémica; y 27 muestras de otras patologías involucrados en el diagnóstico diferencial de la enfermedad, micosis ^12^ y la enfermedad de Chagas ^15^.

Por otro lado, se excluyeron las muestras de sueros procedentes de pacientes embarazadas, inmunosuprimidos o aquellas muestras hemolizadas o son signos de contaminación.

### Análisis estadístico

Se determinó medidas de frecuencia y porcentajes, se construyeron tablas de contingencia con los resultados obtenidos, se evalúo el rendimiento diagnóstico de los antígenos de *Leishmania (V.) braziliensis* y *Leishmania (V.) peruviana* frente al método de referencia EMD en caso de los cutáneos y el método de IFI en caso de mucosos, mediante la distribución de X^2^ con ajuste de Bonferroni.

Se calcularon los estimadores e intervalos de confianza al 95% de las medidas de rendimiento diagnóstico como sensibilidad, especificidad ^16^, área bajo la curva ROC, valor predictivo positivo, valor predictivo negativo, *likelihood ratio* positivo, *likelihood* ratio negativo para cada una de los antígenos. Los análisis fueron realizados con el programa estadístico Stata v17,1 (Stata Corporation, College Station, Texas, USA) empleando un nivel de significancia de 0,05.

## RESULTADOS

En el presente estudio, el 87,2% de las muestras correspondieron a pacientes con LTA confirmada por el EMD, mientras que el 12,8% lo conformó muestras de pacientes sin sospecha para la enfermedad (Material suplementario Tabla S1).

Se observó en la electroforesis SDS-PAGE que los antígenos solubles y de ES presentaron un perfil proteico desde aproximadamente 15 kDa hasta los 250 kDa, tanto en *Leishmania braziliensis* como en *Leishmania peruviana* (Material suplementario Figura S1).

De la evaluación frente a los sueros controles se obtuvo que, los antígenos solubles de *Leishmania (V.) braziliensis* presenta una sensibilidad del 87,7% (IC95%: 80,8-92,8) y especificidad del 100% (IC95%: 85,2-100), mientras que, entre los grupos presentó una sensibilidad del 96% (IC95%: 90,1-98,9) para la forma cutánea y 60% (IC95%: 40,6-77,3) en mucosos, además presentó reactividad cruzada, 20% frente a sueros de pacientes con la enfermedad de Chagas y 8,3% frente a pacientes con micosis.

Por su parte, el antígeno soluble de *Leishmania (V.) peruviana,* presentó una sensibilidad del 92,3% (IC95%: 86,3-96,2) y especificidad del 95,7%; entre grupos tuvo una sensibilidad del 91% para la forma cutánea, 97,7% para la mucosa, como se detalla en la Tabla 1, y una reactividad cruzada de 33,3% y 50% para la enfermedad de Chagas y micosis respectivamente (Tabla 2).

**Tabla 1:** Rendimiento diagnóstico de los Ag. Solubles y de excresión/secreción de *Leishmania braziliensis* y *Leishmania peruviana.*

		LbAS (IC 95%)	LpAS (IC 95%)	LbAES (IC 95%)	LpAES (IC 95%)
*Leishmaniasis tegumentaria* (Cutánea y mucosa)	Sensibilidad	87,7 (80,8; 92,8)	92,3 (86,3; 96,2)	16,9 (10,9; 24,5)	43,8 (35,2; 52,8)
	Especificidad	100 (85,2; 100)	95,7 (78,1; 99,9)	100 (85,2; 100)	82,6 (61,2; 95,0)
	Área bajo la curva ROC	0,954 (0,91; 0,97)	0,94 (0,89; 0,99)	0,58 (0,55; 0,62)	0,63 (0,54; 0,72)
	Valor predictivo positivo	100 (96,8; 100)	99,2 (95,5; 100)	100 (84,6; 100)	93,4 (84,1; 98,2)
	Valor predictivo negativo	59,0 (42,1; 74,4)	68,8 (50,0; 83,9)	17,6 (11,5; 25,2)	20,7 (12,9; 30,4)
	*Likelihood ratio* positivo	41,95 (2,7; 652,06)	21,23 (3,12; 144,46)	8,24 (0,52; 131,36)	2,52 (1,01; 6,27)
	*Likelihood ratio* negativo	0,13 (0,08; 0,2)	0,08 (0,04; 0,15)	0,85 (0,77; 0,93)	0,68 (0,53; 0,87)
*Leishmaniasis cutánea*	Sensibilidad	96,0 (90,1; 98,9)	91,0 (83,6; 95,8)	1,0 (0,0; 5,4)	35,0 (25,7; 45,2)
	Especificidad	100 (85,2; 100)	95,7 (78,1; 99,9)	100 (85,2; 100)	82,6 (61,2; 95,0)
	Área bajo la curva ROC	0,98 (0,96; 1,00)	0,93 (0,88; 0,98)	0,50 (0,50; 0,51)	0,59 (0,50; 0,68)
	Valor predictivo positivo	100 (96,2; 100)	98,9 (94,1; 100)	100 (2,5; 100)	89,7 (75,8; 97,1)
	Valor predictivo negativo	85,2 (66,3; 95,8)	71,0 (52,0; 85,8)	18,9 (12,3; 26,9)	22,6 (14,2; 33,0)
	*Likelihood ratio* positivo	45,86 (2,95; 712,46)	20,93 (3,08; 142,46)	0,71 (0,03; 16,96)	2,01 (0,79; 5,10)
	*Likelihood ratio* negativo	0,05 (0,02; 0,11)	0,09 (0,05; 0,18)	1,01 (0,94; 1,07)	0,79 (0,62; 1,00)
*Leishmaniasis mucosa*	Sensibilidad	60,0 (40,6; 77,3)	96,7 (82,8; 99,9)	70,0 (50,6; 85,3)	73,3 (54,1; 87,7)
	Especificidad	100 (85,2; 100)	95,7 (78,1; 99,9)	100 (85,2; 100)	82,6 (61,2; 95,0)
	Área bajo la curva ROC	0,80 (0,71; 0,89)	0,96 (0,91; 1,00)	0,85 (0,77; 0,93)	0,78 (0,67; 0,89)
	Valor predictivo positivo	100 (81,5; 100)	96,7 (82,8; 99,9)	100 (83,9; 100)	84,6 (65,1; 95,6)
	Valor predictivo negativo	65,7 (47,8; 80,9)	95,7 (78,1; 99,9)	71,9 (53,3; 86,3)	70,4 (49,8; 86,2)
	*Likelihood ratio* positivo	28,65 (1,82; 451,69)	22,23 (3,27; 151,35)	33,29 (2,12; 522,18)	4,22 (1,69; 10,54)
	*Likelihood ratio* negativo	0,41 (0,27; 0,63)	0,03 (0,01; 0,24)	0,31 (0,18; 0,53)	0,32 (0,17; 0,60)

IC 95%=Intervalo de confianza al 95%. LbAS: antígeno soluble de *Leishmania braziliensis*; LpAS: antígeno soluble de *Leishmania peruviana*;

LbE/S: antígeno de excresión/secreción de *Leishmania braziliensis*: LpE/S: antígeno de excresión/secreción de *Leishmania peruviana.*

**Tabla 2:** Reactividad cruzada de los antígenos solubles y antígenos ES de *Leishmania braziliensis* y *Leishmania peruviana.*

		Resultado		E (IC 95%)	Reactividad cruzada
		VN	FP		PFP (FP/n)
Enfermedad de Chagas (n=15)					
	LbAS	12	3	80,0 (51,9; 95,7)	20,0
	LpAS	10	5	66,7 (38,4; 88,2)	33,3
	LbAES	15	0	100 (78,2; 100)	0,0
	LpAES	15	0	100 (78,2; 100)	0,0
Micosis (n=12)					
	LbAS	11	1	91,7 (61,5; 99,8)	8,3
	LpAS	6	6	50,0 (21,1; 78,9)	50,0
	LbAES	12	0	100 (73,5; 100)	0,0
	LpAES	12	0	100 (73,5; 100)	0,0
General (n=27)					
	LbAS	23	4	85,2 (66,3; 95,8)	14,8
	LpAS	16	11	59,3 (38,8; 77,6)	40,7
	LbAES	27	0	100 (87,2; 100)	0,0
	LpAES	27	0	100 (87,2; 100)	0,0

*E: especificidad; IC 95%=Intervalo de confianza al 95%. LbAS: antígeno soluble de Leishmania braziliensis; LpAS: antígeno soluble de Leishmania peruviana; LbE/S: antígeno de excreción/secreción de Leishmania braziliensis: LpE/S: antígeno de excreción/secreción de Leishmania peruviana; VN: verdaderos negativos; FP: falsos positivos; PFP: proporción de falsos positivos.*

Asimismo, de la evaluación de la concordancia en la detección de LTA, considerando como criterio de positividad las fracciones entre 50-55 kDa (Figura 1), el antígeno soluble de *Leishmania (V.) braziliensis* presentó un índice kappa de 0,68 y 0,76 para *Leishmania (V.) peruviana*; por otro lado, entre los grupos cutáneos y mucosos, el antígeno soluble de *Leishmania (V.) braziliensis* presentó un índice kappa de 0,9 y 0,76, respectivamente, y en caso de *Leishmania (V.) peruviana,* de 0,56 para los cutáneos y 0,92 para la forma mucosa de la enfermedad (Material suplementario Tabla S2).

**Figura 1:** Comparación del área bajo la curva de los antígenos solubles y de excresión/secreción de *Leishmania (V.) braziliensis y Leishmania (V.) peruviana*. Donde LbT: antígeno soluble de Leishmania *braziliensis*; LpT: antígeno soluble de *Leishmania peruviana*; LbE/S: antígeno de excresión/secreción de *Leishmania braziliensis*: LpE/S: antígeno de excresión/secreción de *Leishmania peruviana*: AUC: área bajo la curva.

En cuanto al rendimiento diagnóstico de LTA, el antígeno LbAS alcanzó un área bajo la curva de 0,95; para la forma cutánea 0,98 y 0,80 para la mucosa; mientras que, el LpAS alcanzó un área bajo la curva de 0,94 en la detección de LTA; 0,93 para la forma cutánea y 0,96 para la mucosa, como se detalla en la Tabla 1 y el material suplementario Figura S3.

## DISCUSIÓN

En el presente estudio, el método de Inmunoblot, ha permitido entre otros, evidenciar la basta diversidad de proteínas que presentan *Leishmania* spp ^17^^,^^18^*,* la interacción antígeno - anticuerpo mediante una reacción inmunoenzimática, así como la identificación de potenciales biomarcadores que podrían mejorar al diagnóstico serológico de la enfermedad ^19^.

Estudios relacionados empleando antígeno obtenido de *Leishmania major* MRHO/IR/75/ER, lograron determinar que la proteína con mejor rendimiento en la detección de anticuerpos anti-*Leishmania* fue 63 kDa, esta fracción presentó una sensibilidad de 96,7% y una especificidad del 70% ^20^, mientras que, Brito *et al*., (2000) empleando proteínas de *Leishmania braziliensis* MHOM/BR/75/M2903 frente a sueros de pacientes confirmados, reportaron como proteínas inmunogénicas aquellas de peso molecular 30 y 27 kDa, los cuales fueron reconocidos en el 88% y 91% de los casos, la fracción proteica de 48 kDa en el 70%, y aquellas de 60 kDa y 66 kDa en menos del 35% de las muestras. Considerando la visualización de estas fracciones proteicas como criterio de positividad en la detección de anticuerpos anti-*Leishmania* reportaron una sensibilidad del 91% y especificidad del 100% ^21^; y del 76,9% y 100% respectivamente empleando antígenos de *Leishmania (V.) braziliensis* MHOM/BR/1987/M11272 considerando las proteínas de peso molecular 42, 58 y 63 kDa como criterio de positividad ^17^.

En el presente estudio, las proteínas inmunogénicas con mejor rendimiento en la detección de LTA fueron entre 50-55 kDa que alcanzaron una sensibilidad del 87,7% y especificidad del 100% empleando *Leishmania (V.) braziliensis,* y del 92,3% y 95,7% respectivamente en *Leishmania (V.) peruviana.* Por el contrario, las proteínas 63, 42, 30 y 27 kDa previamente reportadas presentaron frente a las muestras incluidas en el estudio, una frecuencia por debajo del 15% frente a sueros cutáneos y mucosos.

Estas diferencias en el carácter inmunogénico de *Leishmania* probablemente esté relacionado con las características intrínsecas de cada especie e inclusive dentro de la misma especie, si bien los estudios previos fueron realizados empleando las cepas de *Leishmania braziliensis* MHOM/BR/75/M2903 y MHOM/BR/1987/M11272, ambas corresponden a aislamientos procedentes de Brasil, por el contrario, este estudio se desarrolló empleando la cepa MHOM/PE/84/LC53 aislada en Perú. Cabe precisar que, estudios en *Leishmania braziliensis* han demostrado la divergencia genética inclusive en aquellas que fueron aisladas dentro de un mismo espacio geográfico ^22^ cada una de ellas con características claramente diferenciables gracias a las metodologías actuales. Razón por el cual el carácter inmunogénico observado en el presente estudio, podría estar relacionado al componente genético de la especie.

Con respecto a los antígenos ES, estos presentaron una elevada especificidad, pero limitada sensibilidad, resultado que se asemeja a lo reportado por Longoni *et al*., (2014) quienes a partir de cepas de *Leishmania amazonensis* y *Leishmania peruviana* y empleando antígenos solubles reportaron una sensibilidad de 21,6% y 56,9% respectivamente, mientras que, al emplear los antígenos secretados de superóxido dismutasa de hierro en ambas especies la sensibilidad fue del 82,4% y 11,8%, respectivamente ^23^.

Actualmente, la inmunofluorescencia indirecta es el método ampliamente utilizado en el diagnóstico serológico de la enfermedad, presenta una sensibilidad y especificidad variable que depende de factores como el tiempo de evolución de la enfermedad y la forma clínica de la enfermedad, además de la especie de *Leishmania* empleada como antígeno, en ese sentido, empleando promastigotes de *Leishmania infantum* y sueros de pacientes inmunosuprimidos con leishmaniasis visceral, el método de IFI presento una sensibilidad del 79,4% y especificidad del 99,2% ^8^; por el contrario, en la forma tegumentaria, la sensibilidad del método en la detección de LTA es 90% y una especificidad entre 62% al 100% ^8^^,^^19^.

En cuanto a la reactividad cruzada evaluada frente a sueros de pacientes con paracocidiomicosis, toxoplasmosis y la enfermedad de Chagas, empleando *Leishmania amazonensis,* fue del 23,4%; 0% y 70% respectivamente, mientras que, empleando promastigotes de *Leishmania braziliensis* fue del 23,4%; 12,5% y 80% ^24^. En el Perú, considerando muestras de pacientes confirmados para la enfermedad con un tiempo de evolución mayor a 2 meses y empleando promastigotes de *Leishmania braziliensis* como antígeno, el Laboratorio de Referencia Nacional de Metaxénicas y Zoonosis Parasitarias reporta una sensibilidad del 83.95% (IC95 75,34 - 92,56), especificidad del 82,76% (IC95%: 72,2-93,3) y reactividad cruzada principalmente con la enfermedad de Chagas (datos no publicados).

Por otro lado, el bajo número de muestras procedentes principalmente de la forma mucosa, el limitado número de muestras procedentes de otras patologías, así como la ausencia de control negativo procedente de área endémica, representan la principal limitación del estudio, debido a que no permitió identificar las fracciones proteicas que interactúan en este grupo de muestras lo que podría representar un sesgo, por lo que recomendamos continuar la evaluación de estos antígenos empleando un mayor número de muestras.

En conclusión, en el presente estudio se evidenció que las proteínas de 50-52 y 55 kDa aproximadamente del antígeno soluble de *Leishmania braziliensis* presenta una sensibilidad del 88% y especificidad del 100%, mientras que, la sensibilidad y especificidad encontrada a partir de los antígenos solubles de *Leishmania peruviana* fue del 92% y 96% respectivamente.

De acuerdo a los hallazgos del estudio, es importante el desarrollo de la validación del método de Inmunoblot empleando los antígenos solubles de *Leishmania braziliensis* y *Leishmania peruviana,* además recomendamos el desarrollo de estudios orientados a la caracterización y análisis de las regiones inmunogénicas reportadas a fin de continuar con el desarrollo de proteínas recombinantes y sintéticas, orientados a mejorar la eficiencia del diagnóstico serológico de la enfermedad.
